# When health services are powerless to prevent suicide: results from a linkage study of suicide among men with no service contact in the year prior to death

**DOI:** 10.1017/S1463423619000057

**Published:** 2019-06-11

**Authors:** Sharon Mallon, Karen Galway, Janeet Rondon-Sulbaran, Lynette Hughes, Gerry Leavey

**Affiliations:** 1 Faculty of Wellbeing, Education and Language Studies, Open University, Milton Keynes, Bucks, UK; 2 School of Nursing, Queen’s University, Belfast, UK; 3 Bamford Centre for Mental Health, University of Ulster, Coleraine, UK

**Keywords:** general practice, help-seeking, men’s health, mental health, suicide

## Abstract

**Aims:**

To investigate cases of suicide in which there was no healthcare contact, by looking at history of help-seeking and evidence of previous mental health vulnerability. To identify any life events associated with suicide for which individuals did not seek help.

**Background:**

Previous research has suggested that non-consultation is the main barrier to suicide prevention among men. Estimates suggest approximately 22% of men who die by suicide have not consulted their GP in the year before their death. Little is known about the lifetime pattern of engagement with services among these individuals and whether or not this may influence their help-seeking behaviour before death.

**Methods:**

Coroner records of suicide deaths in Northern Ireland over 2 years were linked to general practice (GP) records. This identified 63 individuals who had not attended health services in the 12 months before death. Coroner’s data were used to categorise life events associated with the male deaths. Lifetime mental health help-seeking at the GP was assessed.

**Findings:**

The vast majority of individuals who did not seek help were males (*n*=60, 15% of all suicide deaths). Lack of consultation in the year before suicide was consistent with behaviour over the lifespan; over two-thirds had no previous consultations for mental health. In Coroner’s records, suicides with no prior consultation were primarily linked to relationship breakdown and job loss. These findings highlight the limitations of primary care in suicide prevention as most had never attended GP for mental health issues and there was a high rate of supported consultation among those who had previously sought help. Public health campaigns that promote service use among vulnerable groups at times of crisis might usefully be targeted at those likely to be experiencing financial and relationship issues.

## Background

Mental health training for GPs is consistently highlighted as a clear means of improving the effectiveness of suicide prevention (Rutz *et al*., 1989; Pfaff *et al*., 2001). The value placed on primary care services comes in part from evidence that individuals who die by suicide are more likely to have contact with primary care providers than mental health specialists during the final year of life (Pearson, 2009). One recent study reported that 63% of individuals consulted their GP at least once in the year before death (NCISH, 2014). GPs have thus been identified as key enablers to allow clinical services to help prevent suicide (Mann *et al*., 2005; O’Connor *et al*., 2013).

However, there are a number of factors that need to be considered when reflecting upon the role of primary care on suicide prevention. In particular, two recent qualitative studies have challenged the assumption that attendance at the GP can automatically reduce suicide by highlighting significant and underrated challenges to the assessment and treatment of suicide risk during primary care consultations. For example, Michail and Tait (2016) revealed wide variation in the ways GPs understand and operationalise suicide risk in their practice. However, Chandler *et al.* (2016) highlighted the struggle GPs have in defining suicidality, and their difficulties in distinguishing self-harm from ‘truly suicidal’ practices (Chandler *et al.*, 2016). These new findings connect with the long-standing and considerable variation in the consultation rates reported in studies of attendance at primary care before death by suicide (Luoma *et al*., 2002; Stene-Larsen and Reneflot, 2017) to demonstrate how the relationship between attendance at the GP and subsequent risk of suicide is not straightforward.

In addition, the literature shows attendance rates are heavily influenced by the population studied, particularly as contact varies with gender and age. For example, one study estimated that 100% of women and 78% of men had contact with primary care services within 1 year of their suicide (Luoma *et al.*, 2002). Another showed that only 56% of male suicides aged 15–39 has seen their GP within the 3 months before their death (Stanistreet *et al.*, 2004). Crucially, it is not known how non-consultation behaviour before suicide fits with the lifetime pattern of help-seeking among these individuals. Rates of non-consultation are important because assessment clearly requires those at risk of suicide to initiate contact with services. The ability of GPs to prevent suicide among certain groups, for example young men, may be limited by their non-attendance. This represents a particular challenge to current suicide prevention strategists. Men who do not access services are one of the key target groups listed in the UK’s National Health Service (NHS) Mental Health Five-Year Forward Plan, which aims to reduce suicide by 10% in people aged over 18 by 2021 (The Mental Health Taskforce, 2016). The identification of this group follows previous assertions that non-consultation remains the main barrier to suicide prevention, and that the higher rate of suicide among men may be partly attributed to the lower rate of consultation among this population (Biddle *et al*., 2004). It is estimated that suicide risk is increased 67% in those who are non-consulters at the GP (NCISH, 2014).

In light of this, suicide prevention research has acknowledged that we particularly need to develop approaches that encourage men to recognise the value of contacting their GP for support to manage emotional distress (Stanistreet *et al*., 2004). Despite this, primary care help-seeking among men who die by suicide has rarely been examined in detail (Luoma *et al*., 2002). Until recently, studies including the National Confidential Inquiry in the UK have focussed on suicide among those already referred to psychiatric services (Pearson *et al*., 2009). Other authors such as Owens *et al.* (2003) have focussed on suicide outside of mental health services but included those who attended primary care. Studies that focus on men who did not access their GP or psychiatric services in the final year of life are particularly sparse (Luoma *et al*., 2002); as a result, issues connected to suicides among this group are poorly understood.

The focus on psychiatric service contacts in the literature in part reflects the fact that mental illness has long been recognised as an important risk factor for suicide (Joiner, 2010). However, the role of social and interpersonal factors in contributing to suicide has been increasingly highlighted (Owens *et al*., 2003; Samaritans, 2013). Furthermore, links have recently been made between austerity measures in the UK, unemployment and suicide (Webb and Kapur, 2015). In addition, GPs report that psychosocial problems play a part in approximately 20% of all consultations (Verhaak, 1995). However, cross-disciplinary studies that explore how social circumstances link to individual action and help-seeking have not been forthcoming.

As a reflection of these gaps in the literature, in this study we use innovative sociological mixed methods to extricate potential opportunities for identifying and targeting those individuals who die by suicide outside primary care. The specific aims of this study were to:

- investigate cases of suicide in which there was no recent health service contact;
- identify issues associated with suicide for which individuals did not consult their GP;
- examine individuals with a prior history of consultation for mental health issues in which there was no primary care consultation before suicide.

## Method

### Study design

This is a cohort study of all deaths by suicide over a 2-year period from 2007 to 2009 in Northern Ireland (NI) incorporating sociological autopsy methods. This method accepts that suicides are multi-factorial and can result from a dynamic combination of situations; it thus allows analysis to focus on and explore the clusters of circumstances facing individuals before their death, including social aspects of their lives, in order to bring insights to our understanding about their repertoires of action. Ethical approval for the study was obtained from the NI branch of the UK Office for Research Ethics Committee (ORECNI).

### Sample and data collection

Examination of Coroner’s records identified 325 males and 78 females who died by suicide. Data were extracted from reports collated for the Coroner that included statements by family members and friends in connection with the suicide. Two researchers thematically coded the dominant issues associated with the suicide using the sociological autopsy method (Scourfield *et al*., 2012). Coroner records were then linked to GP records for the individuals who had died. Records of lifetime help-seeking were collated using SPSS v10. Further methodological details are reported elsewhere (Leavey *et al.*, 2016). Individuals were categorised according to service contacts in the last 12 months of their lives. We recorded quantitative and qualitative data on prior consultations for mental health, including details of the presenting issue, treatment offered, evidence of engagement and/or disengagement with treatment, and any other relevant circumstances recorded by their practitioners.

### Data analysis

In this paper, we focus on those individuals who had not consulted services for any reason during the last 12 months of life. We analysed their previous help-seeking across the lifespan, focussing on consultations for mental health problems. Issues associated with any prior mental health consultations were thematically coded using the sociological autopsy method (Scourfield *et al*., 2012). In order to ensure consistency in coding across all areas at data collection stage, inter-rater reliability was carried out across 10 initial cases (see section on Coroner’s data for more detail of additional coding). Statistical analyses were conducted using SPSS v15. Qualitative notes were made in ‘Microsoft Word’.

## Results

### The sample

From the 2-year cohort of suicide deaths (*n*=403), 366 of the primary care health records were matched (91%). Unmatched cases (*n*=37) included missing or unavailable GP records and deaths of people not registered with a GP in NI. In these cases, we were able to use Coroner’s records to determine that 31 of these individuals had seen the GP in the last year of their life. They are therefore included in the overall analysis.

Overall, we identified 60 men and 3 women who had no contact with GP or psychiatric services in the final year of life. This accounted for 15% of the whole cohort, 18% of men and 4% of women. Given the gender bias and the small proportion of all female deaths in this group, we focus here only on the male individuals who died over this 2-year period. A detailed analysis of female deaths has been undertaken in a separate paper (Mallon *et al.*, 2016).

**Figure 1:**
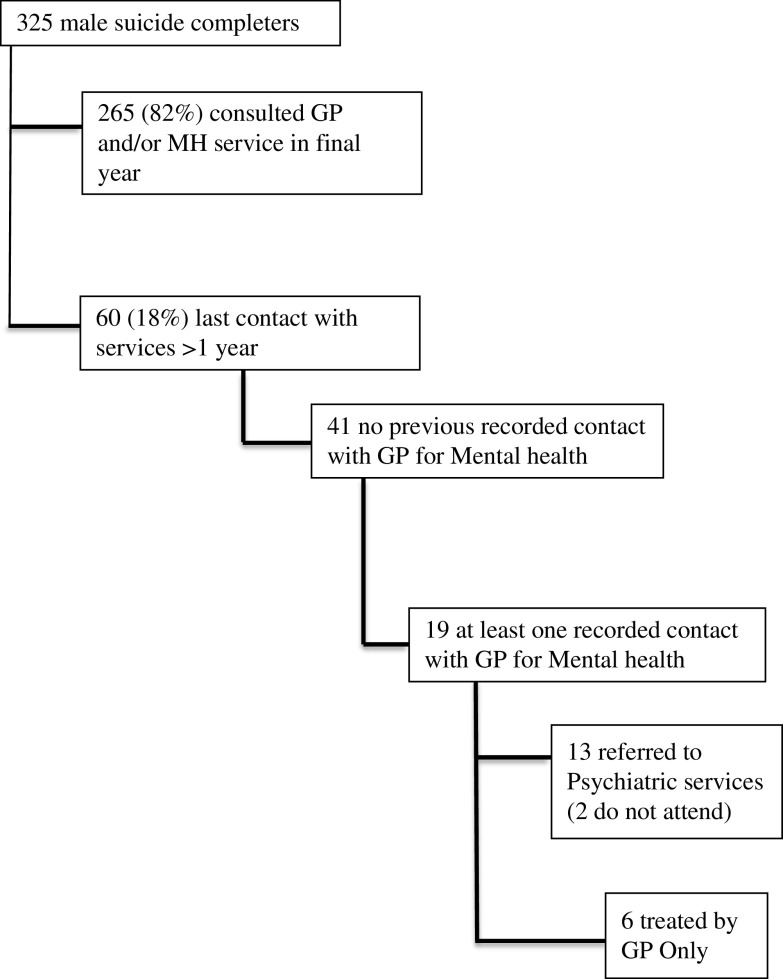
Help-seeking pathways before the suicide across the male cohort

Figure 1 illustrates help-seeking pathways before the suicide across the whole male cohort (*n*=325). Table 1 indicates the percentage of men who consulted by age. Those individuals who had not consulted had a mean age of 34 (SD=15), a median age of 31 and an age range of 11–83 years. Those who had sought help had a mean age of 40 (SD=16), a median age of 39 and an age range of 12–83 years. Those in younger age categories were less likely to have sought help compared to those in the older age categories. For example, 24% (*n*=16) and 20% (*n*=29) of those aged under 25 and 25–44 years, respectively, had not consulted; whereas in the 45–64 age range this figure was slightly lower at just 14% (*n*=13). In contrast, only 8% (*n*=2) of those aged over 65 had not attended the GP. These differences between attenders and non-attenders in terms of age range did not reach statistical significance [*P*=0.06 (χ^2^ excluding >65 s due to small base numbers)]. A lower proportion of those who were not in paid employment, including those who had retired, sought help from the GP relative to those who were in paid employment (12.8%, *n*=21 and 19.9%, *n*=33, respectively). However, statistical analysis in relation to employment, geographical location and relationship status demonstrated no significant differences. Given the focus of this study, we have excluded those men who sought help in the last 12 months of life from the remainder of the analysis reported here.

**Table 1:** Relationship between male age and attendance in the last 12 months

Age group (%)		<25	25–44	45–64	>65	Total
Attendance in L12	Yes	50 (18.9)	115 (43.4)	77 (29.1)	23 (8.7)	265 (100)
Attendance in L12	No	16 (26.7)	29 (48.3)	13 (21.7)	2 (3.3)	60 (100)
Total		66 (20.3)	144 (44.3)	90 (27.7)	25 (7.7)	325 (100)

#### Case note data

*Characteristics of those who did not consult.* GP records of the 60 men who did not consult in the year before their death demonstrated very infrequent consultations across their entire lifespan. In 68% (*n*=41) of these cases medical records showed the men had never previously consulted their GP for mental health (Figure 1).

*Previous mental health consultations.* There were 19 men who had attended the GP with mental health difficulties in the past. We undertook detailed analysis of those 19 men and found six men had been managed by the GP alone with the remaining 13 referred to psychiatric services; two of these men did not attend their appointment. Records showed that eight of the 19 men who had previously attended for mental health difficulties had been accompanied by another individual; in all eight cases an adult female had accompanied the patient, for four the consultations occurred during childhood or adolescence and in the remaining four cases the individual had attended as an adult but was accompanied at the appointment by a female relative. Table 3 illustrates diagnostic categories based on the previous mental health consultation.

We categorised all previous consultations for mental health according to their outcome. In 10 of the 19 cases, the previous episode of help-seeking could be characterised as successful in that the individual had engaged with the treatment offered and as far as medical records were able to indicate, the issue resolved after the intervention. In contrast, in the remaining nine cases, records suggest the treatment offered had failed to resolve the presenting issue. The men were noted to have refused the treatment offered (*n*=2) or had quickly disengaged from services by not attending follow-up appointments (*n*=7). These cases included three of the eight cases, where help sought had been undertaken with the support of an accompanying relative.

#### Coroner’s data

*Issues connected with suicide outside services.* Table 2 presents the dominant issues presented to the Coroner in relation to the suicides of the 60 men who did not seek help, categorised using the sociological autopsy method (Scourfield *et al*., 2012). As indicated earlier, at data collection stage, two researchers thematically coded relevant issues associated with each individual case. Across the 60 cases under discussion here, a dominant theme was identified following a rigorous discussion of each individual’s death. Emergent themes across the cases were then easily grouped together through discussion of all the issues identified across the cohort, with a particular focus on those identified through previous sociologically driven work (Scourfield *et al*., 2012). In 30% of cases, relationship issues, predominantly relating to problems with an intimate partner, were described as being related to the suicide. The remaining deaths were broadly distributed across a range of issues including debt/job loss. Explanations categorised as ‘depressive personality’ refer to those individuals who were described by family members as experiencing low mood for some time but who had not sought treatment or been given a diagnosis. Problems categorised as ‘shame’ refer to emotional issues reported in association with alleged criminal activities. Those classified as ‘impulsive’ are those in which relatives reported being unaware of any particular source of distress.

**Table 2:** Diagnosis of those men who had previously sought help for mental health

No diagnosis noted	10
Depressive disorder	3
Psychological disorders – other	2
Anxiety disorder	2
Acute/chronic alcohol abuse	1
Posttraumatic stress disorder	1

**Table 3:** Factors associated with suicide as presented to the Coroner

	Total *n* (%)
Relationship breakdown	18 (30)
Debt/job loss	11 (18)
Depressive personality	9 (15)
Impulsive/no trigger identified	8 (13)
Trauma	8 (13)
Shame	5 (8)
Related to another suicide	1 (2)
Grand total	60 (100)

Triangulation of the GP and Coroner data demonstrated that six of the 19 men who had previously attended the GP in connection with a particular life event eventually had this issue recorded in the Coroner’s file in connection with their suicide. For example, one man had attended in relation to anxiety following a robbery. Although it had been a few years since this event, his relatives mentioned to the Coroner that he had never fully recovered. Similarly, another man sought help in relation to the death of his mother, years later it was mentioned to the Coroner as a factor in his death. Notably, only one of the men classified as having had a successful help-seeking pathway had the same issue mentioned to the Coroner at the time of death. The remaining five men were classified as having a failed episode of help-seeking in relation to this issue. In all of these cases, there were lengthy gaps between the incident and the eventual death.

## Discussion

### Summary of the main findings

Our study indicates that 18% of men and 4% of women who died by suicide over a 2-year period sought no support from the GP in the year before their death. The rate of non-consultation among men reported here is in accordance with other studies in which it was reported to be around 18–19% (Luoma *et al*., 2002; NCISH, 2014). However, it conflicts with a recent Northern Irish study in which it was reported that only 3.5% of males had not attended services in the last year of life; the authors suggested their figures showed disengagement from services (O’Neill *et al*., 2014). We argue that this variation is likely to relate to varying data collection methods as the authors relied upon medical information recorded within the Coroner’s data when calculating the last service interaction of the male sample. Our figure benefits from triangulation through record linkage to GP records, with Coroner’s data providing only supplementary information when GP records were not available. Therefore, we would conclude that studies relying on Coroner’s data alone to estimate contact with services before suicide may result in significant underestimation and should be interpreted with caution.

Studies have consistently demonstrated lower consultation rates for mental health among men, compared to women (Luoma *et al*., 2002). Our findings strongly reinforce the idea that suicide outside of primary care is almost exclusively a male issue. We make a unique addition to the literature by demonstrating that the lack of help-seeking by these men in the year before their death was consistent with their behaviour across their entire lifespan: over two-thirds of the men who did not consult in the year before their death had never sought help for matters related to mental health. The social behaviour model of help-seeking suggests differences in rates of male and female help-seeking implies that men are largely prohibited from attending the GP by characteristics of their gender (Aday and Andersen, 1974). This supposition is supported by our data as we demonstrated that the lack of help-seeking in the year before their death was largely consistent with behaviour across the lifespan and was not significantly affected by employment or relationship status or geographical location. Those who had sought mental health support more than 1 year ago were commonly accompanied by a supportive female and most were not diagnosed with a mental health problem. Perhaps contrary to expectations in relation to the perception of younger men being hard to reach (Rondón *et al*., 2012), we did not find a significant difference in help-seeking according to age.

Research into suicide has long tended to focus on psychological and psychiatric issues (Hjelmeland and Knizek, 2010). This focus has come under increased scrutiny, with one report by the Samaritans calling for an increase in the recognition of the contribution social issues that can make to suicide (Samaritans, 2013). In this paper, we addressed this concern by using a sociological autopsy approach to classify the dominant issues reported by family members to the Coroner in connection with the suicides of men who did not seek help in the last year of their life. We were able to determine that family members associated the suicides predominantly with relationship breakdown, debt or job loss and generalised depression. The proportion of suicides linked to issues such as debt and relationship breakdown is consistent with that identified in previous research using Coroner’s data (Scourfield *et al*., 2012). In this study we have highlighted how despite family members identifying these issues as causing considerable distress these events did not result in primary care help-seeking. The dominance of social factors may have further meaning as male help-seeking behaviour is known to be affected by self-reported embarrassment (Tedstone *et al*., 2010; Latalova *et al*., 2014). It is also notable that few suicides in this study were coded as being impulsive, in the sense that no explanation could be offered by friends and family. This supports the assertion that suicide on a whim is a rare phenomenon and corroborates the well-established trend that men are likely to disclose distress to family and friends (Joiner, 2010) and further highlights the potential role of lay networks in preventing suicide (Owens, 2011).

### Implications for research and practice

In this study, we sought to inform GPs about the potential limitations of their gate-keeping role in suicide prevention by examining the lives of those who die without consulting their GP. In doing so, we have provided opportunities for identifying and targeting individuals who do not access primary care, but may be at risk of suicide. The findings, therefore, have a number of implications for research and practice.

First, our findings strongly reinforce the notion that suicide outside of primary care is almost exclusively a male issue. The consistency with which the non-consultation rate varies between the genders calls into question the usefulness of studies that combine genders in their analysis. Such studies may misrepresent the help-seeking of both genders and fail to take into account the complex dynamics of gender and help-seeking patterns (Featherstone and Broadhurst, 2003; Mallon *et al.*, 2016).

Second, the 60 men who are the focus of this study did not contact their GP in the year before their death. We know that in 41 cases these men had no pattern of established help-seeking behaviour for mental health problems. At no point in their life had they passed through Goldberg and Huxley’s ‘first filter’, in that they had never previously made a decision to consult for mental health (Goldberg and Huxley, 1992). In an additional eight cases they had done so only with the support of a female relative. As they ultimately died by suicide this would appear to support the notion that the men’s subjective assessment of situations and their recognition of the need for services remains a prohibitive factor in consulting their GP before suicide. We further suggest that given the lifetime pattern of non-consultation among the majority of these men, suicide prevention in this group remains a challenging task, and it is potentially contentious to suggest that the GP is the main gatekeeper to improving suicide prevention and risk assessment among this hard-to-reach group (Biddle *et al*., 2004). In some of these cases, the stressors described by relatives to the Coroner make it difficult to see how in the short term, primary care can contribute to a significant reduction of suicide among this group. The very dominance of factors such as debt and relationship breakdown raises the question of whether the GP is the most appropriate source of help for individuals who may be experiencing vulnerability to suicide because of inequality and other socially-driven stressors. This question is likely to be even more relevant in the current political climate and further highlights the need for doctors and researchers to advocate for action that strengthens non-medical sources of support in the community (British Medical Association, 2016).

Owens *et al.* (2011) suggested it may be possible to improve suicide prevention opportunities at primary care level by strengthening the responses of friends and family, and encouraging their role in facilitating professional help-seeking. This is supported by our findings, as most of the relatives of men who did not seek help were able to identify a triggering issue for the suicide when interviewed by Coroner’s officers. We therefore support Owens’ *et al.* (2011) recommendation that to prevent suicide among those who do not attend services, we need to strengthen lay people’s capacity to recognise, assess, respond to distress and encourage appropriate help-seeking, be this either within primary healthcare, or given the findings of this study other appropriate agencies such as marriage guidance or debt counselling. Further studies that examine how men make decisions to seek help, including the prohibitive processes that stop them from making contact with such services at times of crisis, and how they and their family consider that professional assistance is warranted, is now deemed to be an urgent gap in our knowledge. Prevention services could assist by working to improve public perceptions of what primary care can offer, and promoting the link between GP services and other organisations that provide support through signposting to other relevant agencies. Professionals may also consider how they can play a role in educating the public and wider community on self-management in the acute phases of such distress and how to make decisions about when help from services is warranted. A reframing of the public perceptions of these emotional consequences of largely social (as opposed to health) issues may be warranted if primary care intervention as prevention is to be deemed a realistic option in these cases.

### Limitations

Our study population included all the deaths categorised as suicide by the Coroner over a 2-year period and 93% of these were matched to GP records. Data collection was comprehensive and covered the lifetime help-seeking from the GP. However, there are some limitations to the data presented here. GP records were not available for some of the cohort; in these cases, it was possible to use Coroner’s records to determine if a GP consultation had taken place in the final year of life. This means we can be confident in findings relating to formal help-seeking. Owing to inconsistencies in both the Coroner and GP records in relation to information about support from other sources, such as family members, clergy and charity support (eg, *Lifeline* or *The Samaritans* helplines), we cannot comment on additional support that may have been sought and can only comment on NHS-based GP contacts.

This study relied on data contained within the Coroner’s records to thematically code information offered by families in connection with the suicides. Such data is retrospective in nature; it is therefore dependent on the subjective recall of relatives of the deceased and is situated in the sense that it is collected for the purposes of determining cause of death (Jaworski, 2014; Mallon *et al.*, 2016). However, there are strengths to this approach. In particular, learning more about those issues for which men are reluctant to seek help but which family members consider to be connected to their suicidal actions has the potential to better inform public health campaigns (Hjelmeland and Knizek, 2010).

## Conclusion

We have reiterated the vulnerability of men following relationship breakdown and issues around debt and job loss. These have been previously discussed in the literature (Scourfield *et al.*, 2012) but are gaining increasing prominence as austerity continues to impact. There has been some suggestion that GPs report a lack of time to deal with consultations that have a psychological or social diagnosis (Zantinge *et al.*, 2005). Heightened awareness of the relationship between these issues and suicide is thus warranted. There have been a number of high-profile campaigns aimed at addressing the lack of help-seeking for mental health problems among the male population (Samaritans, 2012). The evidence from this and other studies suggests that mental health promotion strategies could do more than just encourage men to seek help from mental health professionals in response to negative emotions but could specifically target those experiencing particular life events. Perhaps, however, primary care must decide whether to directly attract people who are suicidal into their services for these reasons or invest in supporting their relatives to encourage them to enter and engage with the service.

## Financial support

This research was funded by the Public Health Agency in Northern Ireland.
